# Genetic causes of hypomagnesemia, a clinical overview

**DOI:** 10.1007/s00467-016-3416-3

**Published:** 2016-05-27

**Authors:** Daan H. H. M Viering, Jeroen H. F. de Baaij, Stephen B. Walsh, Robert Kleta, Detlef Bockenhauer

**Affiliations:** 1 Centre for Nephrology, University College London, London, UK; 2 Department of Physiology, Radboud Institute for Molecular Life Sciences, Radboud University Medical Center, Nijmegen, The Netherlands; 3 Paediatric Nephrology, Great Ormond Street Hospital, London, UK

**Keywords:** Magnesium, Homeostasis, Hereditary, Kidney, Distal convoluted tubule, Thick ascending limb of Henle’s loop

## Abstract

Magnesium is essential to the proper functioning of numerous cellular processes. Magnesium ion (Mg^2+^) deficits, as reflected in hypomagnesemia, can cause neuromuscular irritability, seizures and cardiac arrhythmias. With normal Mg^2+^ intake, homeostasis is maintained primarily through the regulated reabsorption of Mg^2+^ by the thick ascending limb of Henle’s loop and distal convoluted tubule of the kidney. Inadequate reabsorption results in renal Mg^2+^ wasting, as evidenced by an inappropriately high fractional Mg^2+^ excretion. Familial renal Mg^2+^ wasting is suggestive of a genetic cause, and subsequent studies in these hypomagnesemic families have revealed over a dozen genes directly or indirectly involved in Mg^2+^ transport. Those can be classified into four groups: hypercalciuric hypomagnesemias (encompassing mutations in *CLDN16*, *CLDN19*, *CASR*, *CLCNKB*), Gitelman-like hypomagnesemias (*CLCNKB*, *SLC12A3*, *BSND*, *KCNJ10*, *FYXD2*, *HNF1B*, *PCBD1*), mitochondrial hypomagnesemias (*SARS2*, *MT-TI*, Kearns–Sayre syndrome) and other hypomagnesemias (*TRPM6*, *CNMM2*, *EGF*, *EGFR*, *KCNA1*, *FAM111A*). Although identification of these genes has not yet changed treatment, which remains Mg^2+^ supplementation, it has contributed enormously to our understanding of Mg^2+^ transport and renal function. In this review, we discuss general mechanisms and symptoms of genetic causes of hypomagnesemia as well as the specific molecular mechanisms and clinical phenotypes associated with each syndrome.

## Introduction

Magnesium is a vital element for the human body and is involved in numerous biological processes. It is the second-most abundant intracellular cation (Mg^2+^) in the human body and is crucial for the function of over 600 enzymes and regulation of the activity of several ion channels, as well as for stabilization of negatively charged molecules such ATP, ADP, RNA and DNA (reviewed in [1]). In order to constantly suffice the body’s requirements for this ion, there is a significant storage capacity for Mg^2+^: an adult human body usually contains about 24 g of Mg^2+^ at any one time [1]. Blood serum only contains a fraction of this, with normal serum Mg^2+^ concentrations [Mg^2+^] ranging from 0.70 to 1.1 mM, which translates to about 60 mg in total. Even though only two-thirds of this is biologically active (the ionized fraction), total serum Mg^2+^ concentrations are still used in daily practice as a measurement of the total Mg^2+^ status of a patient. Accordingly, hypomagnesemia is defined as a serum [Mg^2+^] < 0.70 mM (<1.7 mg/dL) and hypermagnesemia as a serum [Mg^2+^] > 1.1 mM (>2.5 mg/dL). A shortage of Mg^2+^ can have direct consequences, some well-established, others less clear, but it is also associated with several other diseases. Direct consequences or symptoms that might arise from hypomagnesemia are variable in severity and may correlate to the extent and duration of the Mg^2+^ shortage, ranging from leg cramps and tiredness to seizures, coma and eventually death (Table 1). In addition, (severe) hypomagnesemia may have further consequences during pregnancy, as suggested by findings that a Mg^2+^-deficient diet in pregnant mice was able to induce fetal malformations [2]. Conversely, supplementation with magnesium sulfate (MgSO_4_) during pregnancy is a treatment for pre-eclampsia [3], suggesting a role for a relative shortage of Mg^2+^ in this disease too. Lastly, some diseases, such as Parkinson’s disease and diabetes, have merely been associated with low serum Mg^2+^ concentrations (reviewed in [1]). It is not yet clear, however, whether hypomagnesemia is the cause, a consequence or simply an epiphenomenon in these diseases.

**Table 1:** Direct consequences of hypomagnesemia

Direct consequences of hypomagnesemia^a^
Chvostek and Trousseau’s signs
Tiredness
Generalized weakness
Tremor
Paresthesias and palpitations
Hypokalemia
Hypoparathyroidism resulting in hypocalcemia
Chondrocalcinosis
Failure to thrive (in children)
Spasticity and tetany
Seizures
Electrocardiography changes, including prolonged QT interval (especially with concomitant hypokalemia)
Cardiac arrhythmias (especially with concomitant hypokalemia)
Basal ganglia calcifications
Coma
Intellectual disability
Death

^a^Consequences of hypomagnesemia are presented from top to bottom of table in order of increasing severity

Most of our current knowledge about Mg^2+^ homeostasis has been obtained by studying the molecular mechanisms through which genetic mutations cause hypomagnesemia. The aim of this review is to provide an update of the currently known genetic defects in Mg^2+^ homeostasis from a clinical point of view, discussing firmly established knowledge as well as several recently discovered or neglected hereditary hypomagnesemic syndromes.

## Maintaining Mg^2+^ homeostasis

The daily dietary Mg^2+^ intake recommended by the U.S. Institute of Medicine is dependent on age (Table 2). Approximately 30–50 % of the ingested Mg^2+^ will be absorbed by the intestine, although this has been reported to increase to 80 % in cases of Mg^2+^ deficiency. The bulk of the dietary Mg^2+^ is initially absorbed in the jejunum and ileum via paracellular pathways. The remainder can be absorbed by the colonic epithelium, entering the cells via transient receptor potential melastatin type 6 (TRPM6), an essential ion channel and serine/threonine-protein kinase, and probably exiting the cells at the basolateral side by making use of the sodium (Na^+^) gradient and the Na^+^–Mg^2+^ exchanger cyclinM4 (CNNM4). Mg^2+^ subsequently enters the bloodstream to be delivered to cells, excreted by the kidneys or stored in bones. The large skeletal stores (50–60 % of total body Mg^2+^) are in part responsible for keeping the serum Mg^2+^ concentrations constant (reviewed in [1]).

**Table 2:** Recommended dietary allowance of magnesium (Mg^2+^)

Age (years)	RDA for males (mg Mg^2+^/day)	RDA for females (mg Mg^2+^/day)^a^
0-1	NA	NA
1–3	80	80
4–8	130	130
9–13	240	240
14–18	410	360
19–30	400	310
>31	420	320

RDA, Recommended dietary allowance; NA, information not available

^a^For women during pregnancy the RDA is slightly higher

**Fig. 1:**
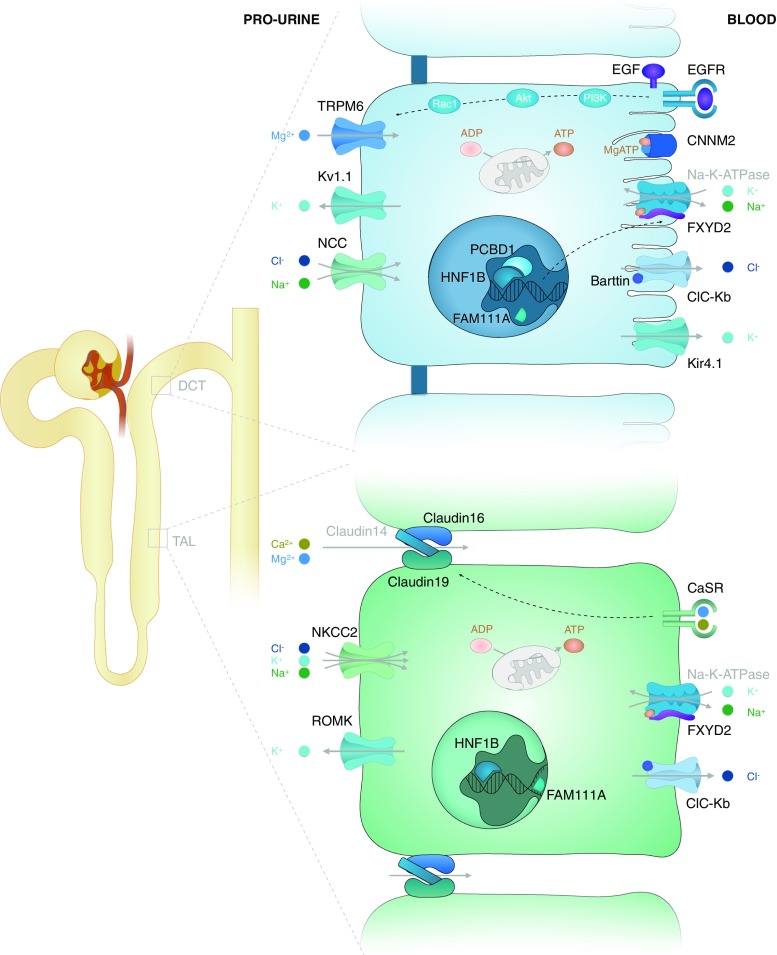
Reabsorption of the magnesium cation (*Mg* ^*2+*^) in the thick ascending limb of Henle’s loop (*TAL*) and distal convoluted tubule (*DCT*). The relevant molecular transport mechanisms of the TAL and DCT are shown. Note that Mg^2+^ is transported via paracellular pathways into the TAL and via transcellular pathways into the DCT. A more detailed explanation of the molecular transport mechanisms can be found in the text. Black text indicates proteins that are mutated in genetic disorders of Mg^2+^ homeostasis. Grey text indicates other proteins

An even more important role in the regulation of Mg^2+^ homeostasis has been given to the kidney. After glomerular ultrafiltration, a mere 10–25 % of Mg^2+^ is reabsorbed by the proximal convoluted tubule (PCT) through paracellular pathways. Next, a further 50–70 % of the filtered Mg^2+^ is reabsorbed via paracellular pathways in the thick ascending limb of Henle’s loop (TAL), where claudins play a key role in regulating paracellular calcium (Ca^2+^) and Mg^2+^ transport [4] (see Fig. 1). Lastly, fine-tuning of the total Mg^2+^ reabsorption takes place in the distal convoluted tubule (DCT), which absorbs the final 5–10 % via transcellular pathways [5]. Essential for this last step is the apical Mg^2+^ channel TRPM6, the same channel that is responsible for Mg^2+^ transport in the large intestine (reviewed in [1]). The activity of this channel and its expression in the membrane are positively regulated through epidermal growth factor (EGF)-activated pathways [6] (see Fig. 1). Finally, also insulin, estrogen, extracellular pH, ATP, oxidative stress and Mg^2+^ itself are all found to be able to influence the magnitude of Mg^2+^ transport in the DCT [1]. Towards the end of this last segment, 95–99 % of filtered Mg^2+^ has been reabsorbed in total, and no further reabsorption takes place beyond this point [7].

## Disturbances of Mg^2+^ homeostasis

Hypomagnesemia is defined as total serum Mg^2+^ concentrations below 0.70 mM (<1.7 mg/dL), while hypermagnesemia is reserved for concentrations above 1.1 mM (>2.7 mg/dL). Symptomatic hypermagnesemia is rare and mostly induced by excessive use of drugs which contain high amounts of Mg^2+^, including laxatives and Epsom salts [1]. Hypomagnesemia on the other hand is more frequent [8–10] and can have several distinct causes. First among these is a prolonged general loss of electrolytes, such as during periods of vomiting, diarrhea or malabsorption [11]. Secondly, several genetic disorders are accompanied by hypomagnesemia (see Table 4). Thirdly, albeit rare in the pediatric population, use of alcohol and certain drugs (Table 3; reviewed in [1]) should be considered. Lastly, contemporary food intake is relatively poor in Mg^2+^ [12] and may therefore contribute to the development of hypomagnesemia.

**Table 3:** Drugs associated with hypomagnesemia

Drugs associated with hypomagnesemia
Diuretics (furosemide, thiazide)
Epidermal growth factor receptor inhibitors (cetuximab)
Proton pump inhibitors (all, such as omeprazole)
Calcineurin inhibitors (cyclosporin A, tacrolimus)
Platinum derivatives (cisplatin, carboplatin)
Antimicrobials (aminoglycosides, pentimidine, rapamycin, amphotericin B, foscarnet)

## Diagnosing renal Mg^2+^ wasting

The most important clinical diagnostic tool for differentiating hypomagnesemia of renal origin from intestinal hypomagnesemia is determination of the fractional excretion of magnesium (FEMg) [13], which can be calculated with the following formula: {([Mg^2+^]_urine_ × [creatinine]_plasma_)/ (0.7 [Mg^2+^]_plasma_ × [creatinine]_urine_)} × 100 %. The factor of 0.7 is included to adjust the total plasma Mg^2+^ concentration to the freely filtered fraction. A FEMg of >4 % in a hypomagnesemic patient is consistent with renal Mg^2+^ wasting, while a patient with a FEMg of <2 % will likely have an extra-renal origin of their hypomagnesemia [13]. However, a FEMg <4 % does not rule out renal Mg^2+^ wasting. First, a low glomerular filtration rate may result in a lower filtered load of Mg^2+^. If the absorptive capacity of the kidney for Mg^2+^ is sufficient to cope with this lower load, the result may be a normal or even low FEMg. By the same mechanism, severe (renal) hypomagnesemia may result in a lower filtered load of Mg^2+^ and thus a normal or low FEMg. To account for these confounding factors, the serum Mg^2+^ levels of hypomagnesemic patients should be increased by means of intravenous Mg^2+^ supplementation before the FEMg is measured [14].

## Hereditary hypomagnesemias

The genetic causes of hypomagnesemia are heterogeneous and comprise both recessive and dominant disorders (Table 4). The localization of the responsible genes is firmly established: all known genes encode proteins expressed in the DCT and/or TAL (Fig. 1). Nevertheless, the exact mechanisms at the molecular level remain to be elucidated for many of these diseases. We therefore propose four categories of genetic causes of hypomagnesemia based on similar manifestation, electrolyte abnormalities and localization. The resulting classes presumably also reflect a common pathophysiological mechanism for each group.

**Table 4:** Genetic causes of hypomagnesemia

Categories/names of disorders^a^	Gene	Protein	OMIM catalog number	Inheritance	Renal tubule segment	Plasma Mg^2+^ concentration (mM)^b^	Estimated incidence or number of known families/patients	Distinctive findings, other than hypomagnesemia^c^
Hypercalciuric hypomagnesemias								Hypercalciuria, nephrocalcinosis
FHHNC type 1	*CLDN16*	Claudin-16	248250	R	TAL	0.49	100s of patients	Polyuria/polydipsia, elevated serum iPTH, renal failure
FHHNC type 2	*CLDN19*	Claudin-19	248190	R	TAL	0.59	10s of patients	Same as FHHNC type 1, plus ocular abnormalities
ADHH Bartter syndrome type 5	*CASR*	CaSR	601198	D	TAL	0.66	100s of patients	Hypocalcemia with normal or low PTH
Bartter syndrome, type 3 (classical type)	*CLCNKB*	ClC-Kb	607634	R	DCT/TAL	0.63	100s of patients	Gitelman-like phenotype possible, rarely nephrocalcinosis
Gitelman-like hypomagnesemias								Hypocalciuria, hypokalemia, metabolic alkalosis
Gitelman syndrome	*SLC12A3*	NCC	263800	R	DCT	0.49	1:40 000	Chondrocalcinosis at older age
Bartter syndrome, type 4	*BSND*	Barttin	602522	R	DCT/TAL	0.60	10s of patients	Prenatal complications, renal failure early in life possible
EAST syndrome	*KCNJ10*	Kir4.1	612780	R	DCT	0.63	26 patients	Sensorineural deafness, seizures, ataxia
IDH	*FXYD2*	γ-subunit of the Na^+^-K^+^-ATPase	154020	D	DCT	0.47	3 families (29 patients)	
ADTKD/RCAD	*HNF1B*	HNF1β	137920	D	DCT	0.69	1:120 000	Renal, genital and pancreatic abnormalities and MODY5 in highly variable combination and presentation
HPABH4D/RCAD-like	*PCBD1*	PCBD1	264070	R	DCT	0.68	23 patients	MODY5-like
Mitochondrial hypomagnesemias								Variable
HHH	*MT-TI*	Mt. tRNA^ile^	500005	Mt	DCT?	0.71	1 family (38 patients)	Hypertension and hypercholesterolemia
HUPRAS	*SARS2*	SARS2	613485	R	TAL?	0.37	2 families (4 patients)	Hyperuricemia, pulmonary hypertension, renal failure and alkalosis
KSS	Mitochondrial deletion	–	530000	Mt	TAL?	0.51	100s of patients	External ophthalmoplegia, retinopathy and cardiac conduction defects
Other hypomagnesemias								Variable
HSH	*TRPM6*	TRPM6	602014	R	DCT	0.20	10s of patients	Neonatal presentation with severe hypomagnesemia
IRH	*EGF*	EGF	611718	R	DCT	0.59	1 family (2 patients)	Intellectual disability
NISBD2	*EGFR*	EGFR	616069	R	DCT	?	1 patient	Severe inflammation of skin and bowel from birth
HSMR	*CNNM2*	CNNM2	613882	D/R	DCT	0.50	7 families (10 patients)	Intellectual disability, seizures
ADH/EA1	*KCNA1*	Kv1.1	176260	D	DCT	0.37	1 family (21 patients)	Episodic myokymia
KCS2	*FAM111A*	FAM111A	127000	D	TAL?	0.46	10s of patients	Impaired skeletal development and hypocalcemic hypoparathyroidism

^a^ADH, Autosomal dominant hypomagnesemia; ADHH, autosomal dominant hypocalcemia with hypocalciuria; ADTKD, autosomal dominant tubulointerstitial kidney disease; EA1, episodic ataxia type 1; EAST, epilepsy, ataxia, sensorineural deafness and tubulopathy; FHHNC, familial hypomagnesemia with hypocalcemia and nephrocalcinosis; HHH, hypertension, hypercholesterolemia and hypomagnesemia; HPABH4D, hyperphenylalaninemia BH4-deficient; HSH, hypomagnesemia with secondary hypocalcemia; HSMR, hypomagnesemia with seizures and mental retardation; HUPRAS, hyperuricemia, pulmonary hypertension, renal failure and alkalotic syndrome; IDH, isolated dominant hypomagnesemia; IRH, isolated recessive hypomagnesemia; KCS2, Kenny−Chaffey syndrome type 2; KSS, Kearns-Sayre syndrome; NISBD2 neonatal inflammatory skin and bowel disease type 2; RCAD, renal cysts and diabetes; TAL, thick ascending limb of Henle’s loop; DCT distal convoluted tubule

^b^Estimated average. To convert mM [Mg^2+^] to mg/dL, multiply by 2.43

^c^iPTH, Intact parathyroid hormone; MODY5, maturity onset diabetes of the young type 5

### Hypercalciuric hypomagnesemias

The hypercalciuric hypomagnesemias are a class of hypomagnesemias in which the ability of the TAL to reabsorb divalent cations is affected. Ca^2+^ and Mg^2+^ reabsorption takes place via paracellular pathways in the TAL and is therefore strongly dependent on the lumen positive transepithelial potential difference. Consequently, disruption of this voltage difference or of the integrity of this paracellular pathway will impair both Ca^2+^ and Mg^2+^ transport (reviewed in [4]). In two of the four types of Bartter syndrome, the lumen positive transepithelial voltage difference is decreased, while mutations in the *CASR*, *CLDN16* and *CLDN19* genes are proposed to interfere with the integrity of the pathway as well as the voltage difference [4]. Compensatory mechanisms in the DCT and other segments, however, may be able to avert hypomagnesemia (in Bartter syndrome type 1 and 2) or hypercalciuria (in Bartter syndrome type 4) [15]. This only leaves Bartter syndrome type 3, which impairs both TAL and DCT function (reviewed in [15]), as meeting the criterion for this group. Clinically, genetic disorders in this group can result in nephrocalcinosis or in chronic kidney disease (CKD), although the incidence and speed of progression differs from one to the other [4, 15].

#### *CLDN16* and *CLDN19* (familial hypomagnesemia with hypocalcemia and nephrocalcinosis)

Recessive mutations in *CLDN16* (encoding claudin-16) and *CLDN19* (encoding claudin-19) are the most frequent cause of hypercalciuric hypomagnesemia [16, 17]. These claudin mutations disrupt the pore selectivity of the tight junction, impairing paracellular Ca^2+^ and Mg^2+^ reabsorption in the TAL (reviewed in [18]). Consequently, patients suffer from hypomagnesemia and its associated symptoms, childhood nephrocalcinosis possibly due to the hypercalciuria and polyuria with polydipsia due to additional sodium (Na^+^) and volume loss [19]. Patients with *CLDN19* mutations will also exhibit ocular anomalies [17]. The renal prognosis for both types is poor, with progressive CKD requiring renal replacement therapy typically in the second or third decade of life [20]. The cause of the CKD is unclear, although nephrocalcinosis may be a contributory factor.

#### *CASR* gain-of-function (autosomal dominant hypocalcemia with hypercalciuria)

Gain-of-function mutations in the gene encoding the calcium sensing receptor CaSR (*CASR*) are associated with hypercalciuric hypocalcemia and occasionally with hypomagnesemia [21, 22]. The two large exofacial lobes of the CaSR act as a peritubular Ca^2+^ and Mg^2+^ sensor in the TAL and other tissues (reviewed in [23]). Gain-of-function mutations, analogous to higher Ca^2+^ concentrations, cause the CaSR to suppress salt reabsorption in the TAL and interfere with the claudin-mediated pore selectivity in the tight junctions (reviewed in [23]). Hypercalciuric hypocalcemia with relative hypoparathyroidism is the most important symptom, which, especially when “mistreated” with vitamin D, can lead to nephrocalcinosis in certain cases (reviewed in [24]). Moreover, in patients with more severe gain-of-function of *CASR*, significant wasting of Mg^2+^, Na^+^, potassium (K^+^) and water can also occur [22]. This has led to the alternative name of Bartter syndrome type V in patients with this severe type of presentation [22].

#### *CLCNKB* (Bartter syndrome type III)

Homozygous or compound heterozygous mutations in *CLCNKB*, which encodes the chloride ion (Cl^−^) channel ClC-Kb, cause Bartter syndrome type III. ClC-Kb is expressed basolaterally in the TAL and DCT, providing a pathway for (Cl^−^) to exit the cell. Mutations in the channel therefore interfere with regulation of intracellular chloride levels and the function of NCC (thiazide-sensitive NaCl cotransporter) and NKCC (Na^+^-K^+^-Cl^−^ cotransporter) (reviewed in [15, 25] and [26]). Patients with mutations in *CLCNKB* often present during the first years of life, suffering from a Bartter-like phenotype, including hypercalciuria and loss of Na^+^, K^+^ and water. When they grow older, however, a shift to a more Gitelman-like phenotype can be observed, with marked hypocalciuria and hypomagnesemia in addition to the loss of Na^+^, K^+^ and water [25, 27]. The *CLCNKB* gene is thus listed under the hypercalciuric as well as under the Gitelman-like hypomagnesemias.

### Gitelman-like hypomagnesemias

The genes from the second group of hypomagnesemias listed in Table 4, the Gitelman-like hypomagnesemias, all encode proteins that are involved in the transport of Na^+^, K^+^ and/or Cl^−^ in the DCT. Adequate transcellular Mg^2+^ reabsorption in the DCT is dependent on the apical membrane potential, which is lumen positive when compared to the cytoplasm (reviewed in [28]). Therefore, Mg^2+^ reabsorption also depends on the intactness of other ion transport processes in the DCT. Alternatively, it has been proposed that atrophy of the DCT segment is responsible for all symptoms [29], although thiazide diuretics do not cause atrophy of the DCT [30]. Regardless of the mechanism underlying the DCT dysfunction, diseases from this group of hypomagnesemias all lead to increased calcium reabsorption along different nephron segments, proximal as well as distal (reviewed in [29]). This obviously results in hypocalciuria. In addition, the DCT dysfunction leads to fluid loss and a tendency to lower blood pressures despite an activated renin–angiotensin–aldosterone system (due to compensation mechanisms) (reviewed in [31]). Lastly, the relatively increased levels of aldosterone force the collecting duct to secrete potassium in exchange for sodium, leading to hypokalemia, which, in turn, leads to alkalosis. In addition, the combination of hypomagnesemia with hypokalemia observed in this group can give rise to a prolonged QT interval and cardiac arrhythmias [32–34], justifying avoidance of drugs prolonging the QT interval [32].

#### *SLC12A3* (Gitelman syndrome)

With an estimated prevalence of 1:40 000 [31], Gitelman syndrome is the most frequent genetic cause of hypomagnesemia. It is caused by recessive mutations in *SLC12A3*, the gene encoding the Na^+^-Cl^−^-cotransporter (NCC) that is expressed on the apical membrane of the DCT [35] (reviewed in [31]). Symptoms are generally absent in the first years of life and only towards the end of the first decade do patients start to report symptoms [36]. Affected individuals can suffer from a range of hypomagnesemia-related symptoms, such as cramps, paresthesias or even cardiac arrest [32, 37]. In addition, they can suffer from the salt and water wasting that is apparent in most Gitelman-like hypomagnesemias, resulting in polyuria, salt craving and thirst [37]. The mechanism by which Gitelman syndrome causes hypomagnesemia is still not fully understood. One explanation can be found in the atrophy of the DCT that has been observed in a mouse model of Gitelman syndrome [29]. Additionally, a reduced apical membrane potential and a reduction in TRPM6 activation or mobilization could play a role. It may be speculated that the reduced apical membrane potential is caused by increased NHE2 (Na^+^/H^+^ exchanger)-mediated Na^+^ reabsorption in the DCT as a means to compensate for the NCC dysfunction.

#### *BSND* (Bartter syndrome type IV)

Barttin, encoded by the *BSND* gene, is expressed in the ascending thin limb, TAL, DCT and inner ear as a subunit of the ClC-Kb and ClC-Ka Cl^−^ channels (reviewed in [15, 26]). Consequently, patients with recessive mutations in *BSND* or digenic mutations affecting both ClC-Kb and ClC-Ka will suffer from profound salt wasting in these three tubule segments as well as sensorineural deafness. In addition to complete deafness, Bartter syndrome type IV can be distinguished from the other types of Bartter syndrome by the initial lack of hypercalciuria. In addition, a significant number of patients will develop CKD (reviewed in [15, 26]). Most important for treatment is the adequate supplementation of fluids and sodium directly after birth, much alike all other types of salt-losing tubulopathies with antenatal presentation [38]. Long-lasting treatment with indomethacin can be considered to prevent failure to thrive and decrease renal salt and water wasting, but it should be realized that this treatment will be less effective than in patients with Bartter types I and II [39] and that this drug can cause renal side effects.

#### *KCNJ10* (epilepsy, ataxia, sensorineural deafness and tubulopathy syndrome)

This syndrome, characterized by epilepsy, ataxia, sensorineural deafness and tubulopathy (referred to as EAST syndrome), is a rare recessive genetic disease affecting the K^+^ channel Kir4.1 encoded by *KCNJ10* [40]. This protein is expressed in several tissues, including the central nervous system, inner ear and basolateral side of DCT cells and possibly also TAL cells (reviewed in [41, 42]). In the kidney it forms a basolateral K^+^ channel that conducts outward K^+^ currents, thus recycling the K^+^ imported by the Na^+^-K^+^-ATPase. To aid in diagnosis, magnetic resonance imaging of the brain might show subtle changes, especially in the dentate nuclei of the cerebellum [41]. Patients show often pronounced ataxia and obligate sensorineural deafness. Lastly, although intellectual abilities seem to lag behind [43], it is difficult to assess the intelligence of these patients due to deafness and ataxia impairing both verbal and written communication [44].

#### *FXYD*2 (isolated dominant hypomagnesemia)

Only three families, all Belgian or Dutch, putatively descendants from a common founder [45], have been reported to carry the hypomagnesemia-causing *FXYD2* mutation. The *FXYD2* gene encodes the γ-subunit of the Na^+^-K^+^-ATPase [46]. The specific dominant mutation causes misrouting of this γ-subunit, thereby preventing the splice variant FXYD2b from assembling with the α- and β-subunit of the Na^+^-K^+^-ATPase [46]. Virtually all patients suffer from muscle cramps; additionally, several other hypomagnesemia-related symptoms can occur [45].

#### *HNF1B* (autosomal dominant tubulointerstitial kidney disease)

Heterozygous mutations in the *HNF1B* gene are associated with a multi-system disorder and considered to be the most common genetic cause of congenital anomalies of the kidney and urinary tract (CAKUT) (reviewed in [47, 48]). The *HNF1B* gene is situated in a region susceptible for genomic rearrangements, resulting in a high frequency of large deletions and de novo gene defects [47]. Mutations in this gene can be detrimental to normal development and function of the kidney, pancreas and genital tract [47], thus giving rise to a highly variable set of symptoms originating from these organs, including CAKUT and maturity onset diabetes in the young (MODY).

Hypomagnesemia is also one of these symptoms, occurring in up to 50 % of affected children [49] and sometimes being the first clinical manifestation [50]. Hypomagnesemia becomes more pronounced with increasing age of the patient and can be missed in affected young children. As a result, *HNF1B* mutations are the most common cause of genetic hypomagnesemia for pediatric nephrologists. The current view is that HNF1B dysfunction leads to inadequate transcription of the *FXYD2* splice-isoform *FXYD2a*, which encodes the γa-subunit of the Na^+^-K^+^-ATPase [51]. The diagnosis is complicated by the large variability in presentation and the lack of a clear genotype–phenotype relationship [47]. If a patient is considered to suffer from HNF1B-ADTKD, the diagnosis should only be rejected if point-mutations as well as deletions and insertions in *HNF1B* have been properly excluded.

#### *PCBD1* (renal cyst and diabetes-like)

Recessive mutations in *PCBD1* had long been identified to be responsible for transient neonatal hyperphenylalaninemia and primapterinuria (HPABH4D) [52], but no other symptoms of this genetic defect had been reported until recently. Re-evaluation of earlier identified patients with this syndrome revealed that hypomagnesemia and MODY5-like diabetes are later manifestations of *PCBD1* mutations [53, 54]. This finding also has implications for the importance of looking for alternative genetic diagnoses (including *PCBD1* mutations) if screening for mutations of *PAH* (phenylalanine hydroxylase) is negative after a positive result for the Guthrie test.

The neonatal phenotype can be explained by the failure of PCBD1 to fulfil its role as an enzyme in the metabolism of aromatic acids. The complications appearing later in life are explained by the additional role of PCBD1 as a dimerization factor for HNF1A and HNF1B, regulating their transcriptional activity (reviewed in [53]). This in turn would influence *FXYD2* transcription, causing hypomagnesemia. It should be noted, however, that the CAKUT phenotype often observed in HNF1B patients is not seen in PCBD1-disease due to a different expression pattern of these two proteins. Lastly, hypokalemia and hypocalciuria have not been reported in these patients. Still, it is placed here with the Gitelman-like hypomagnesemias based on the pathophysiological mechanism.

### Mitochondrial hypomagnesemias

The mitochondrial hypomagnesemias, of which the pathophysiological mechanism is still unexplained, have a highly variable presentation. Their phenotype depends both on the nature of the mutation and the fraction of mitochondria affected in each tissue (reviewed in [55]). Some of the mitochondrial hypomagnesemias are associated with Gitelman-like electrolyte abnormalities [56], while others seem to affect TAL function [57–59]. Physiologically, both have a high energy requirement, although the DCT seems to be a better candidate since it has the most mitochondria [60]. Still needing to be clarified is which function of the mitochondrion is most important in Mg^2+^ homeostasis: is it the ATP that is necessary to drive the Na^+^-K^+^-ATPase and to activate CNNM2 [61]? Or could its role in Ca^2+^-signaling be key here?

#### *MT-TI*, *SARS2*, *POLG1* and mitochondrial deletions/duplications

Impaired mitochondrial function might be associated with hypomagnesemia much more frequently than is currently realized. At least three distinct mitochondrial syndromes are accompanied by hypomagnesemia: (1) deletions in the mitochondrial genome as seen in Kearns–Sayre syndrome [62] (2) recessive mutations in the human gene *SARS2* [57] and (3) mutations in the mitochondrial tRNA^Ile^ gene *MT-TI* causing a syndrome with hypertension, hypercholesterolemia and hypomagnesemia [56]. An additional two cases of patients with hypomagnesemia and other mitochondrial diseases have also been identified (mutations in *POLG1* and the mitochondrial Pearson’s syndrome [63, 64]). Since checking serum Mg^2+^ concentrations is not regular clinical practice, it would be interesting to investigate the genuine frequency of hypomagnesemia in patients with mitochondrial disease.

### Other hypomagnesemias

The remaining disorders associated with hypomagnesemia are classified in this review as “other hypomagnesemias’” due to their heterogenic nature. One of these is caused by mutations in *TRPM6*, which encodes the DCT-specific apical Mg^2+^ transporter TRPM6, resulting in an isolated hypomagnesemia (i.e. no other symptoms except secondary to the hypomagnesemia). Others, such as mutations in *EGF* and *EGFR* affect the activity and expression of this channel [6]. *FAM111A* is the only gene in this group of which the pathophysiological mechanism underlying the hypomagnesemia has received no attention at all.

#### *TRPM6* (hypomagnesemia with secondary hypocalcemia)

Mutations in the gene for the DCT- and colon-specific apical Mg^2+^ channel, *TRPM6*, cause the most profound genetic hypomagnesemia [65, 66]. A measured serum Mg^2+^ concentration as low as 0.2 mM or even lower, as low as immeasurable levels, is not uncommon in these patients [14]. Consequently, patients often present with seizures within the first months of life [14]. A defect in the TRPM6 channel impairs epithelial Mg^2+^ resorption in the colon and DCT, thereby inhibiting uptake and stimulating wasting of Mg^2+^, causing significant hypomagnesemia [67]. The secondary hypocalcemia often observed is probably caused by inhibition of the parathyroid gland by the hypomagnesemia, resulting in low levels of parathyroid hormone and eventually leading to hypocalcemia [68].

#### *CNNM2* (hypomagnesemia with seizures and mental retardation)

*CNNM2* is most highly expressed in the TAL, DCT and brain [69, 70], which explains the combination of hypomagnesemia with additional neurological symptoms (anatomical abnormalities, seizures and intellectual disability) seen in patients with dominant or sometimes recessive *CNNM2* mutations [71]. Although CNNM2 was first thought to be a basolateral Mg^2+^ transporter itself, it is now thought to fulfill the role of intracellular Mg^2+^ sensor by undergoing a conformational change upon binding of Mg-ATP [61]. How this conformational change eventually leads to the observed decrease in Mg^2+^ transport, however, remains unclear, as is the precise basis of the neurological symptoms.

#### *EGF* and *EGFR* (isolated recessive hypomagnesemia)

One mutation in the epidermal growth factor gene (*EGF*) has been associated with a recessive form of hypomagnesemia with an additional neurological phenotype (including intellectual disability) [72]. The only mutation identified to date interferes with proper trafficking of pro-EGF, which is expressed in TAL and DCT cells, resulting in a decreased peritubular concentration of autocrine EGF [72]. Subsequently, the signaling cascade from the EGF receptor (EGFR) to the Akt-mediated activation of Rac1 is turned off, resulting in a decrease of endomembrane trafficking of TRPM6 to the apical surface and decreased Mg^2+^ transport [6]. The intellectual disability on the other hand remains unexplained.

Since *EGF* mutations and cetuximab (an EGFR inhibitor) both cause hypomagnesemia [73], it is not surprising that mutations in *EGFR* have also been found to cause hypomagnesemia. Only one patient has been diagnosed to date with loss-of-function of the EGFR. This mutation resulted in severe symptoms comparable to those observed with full EGFR blockade, including skin rash, inflammation of the lungs and bowel and hypomagnesemia [74].

#### *KCNA1* (autosomal dominant hypomagnesemia)

Intriguingly, only one mutation (identified with linkage in a large pedigree) in *KCNA1* has been associated with hypomagnesemia [75], while all other *KCNA1* mutations known to date cause episodic ataxia type 1 without hypomagnesemia (reviewed in [76]). The *KCNA1* gene encodes the voltage-gated K^+^ channel Kv1.1 [75], which is abundantly expressed in certain neurons as well as on the apical membrane of cells in the DCT ([75] and reviewed in [77]). In neurons, the mutations in KCNA1 impair normal repolarization of the membrane potential, resulting in stress-triggered episodes of ataxia and myokymia [77]. Following the same line of reasoning, one might speculate that genetic defects in Kv1.1 might cause hypomagnesemia by depolarizing the apical DCT membrane [78]. However, this theory does not explain why other *KCNA1* mutations have not been reported to cause hypomagnesemia. Also, how does a voltage-gated K^+^-channel like KCNA1, which is closed and thus non-functional at normal apical membrane potential, cause hypomagnesemia in the first place?

#### *FAM111A*

Mutations in *FAM111A* are associated with two distinct dominant diseases: a perinatally lethal disorder called gracile bone dysplasia (GLCEB) and Kenny–Caffey syndrome type 2 (KCS2) [79]. To date, only KCS2 has been associated with hypomagnesemia [80–85]. Other symptoms of KCS2 include severe proportionate short stature, radiological bone anomalies, eye abnormalities and hypocalcemia owing to hypoparathyroidism (reviewed in [82]). FAM111A has been reported to be a host-range restriction factor [86] and shown to be an important component of the cell’s replication machinery near DNA forks, consistent with its proposed role in cancer and cell survival [87–89]. However, it is unclear how this function would link to hypomagnesemia. Alternatively, it might be speculated that its function is related to the function of *TBCE* [90], the causative gene for the clinically closely related Kenny–Caffey syndrome type 1 [91].

## Treatment

Oral or intravenous Mg^2+^ supplementation is the only treatment available for hypomagnesemia of genetic origin. In the acute situation of a (severely) symptomatically hypomagnesemic patient, intravenous Mg^2+^ supplementation can be very important. For adults, a dose of 8–12 g of MgSO_4_ (containing 0.8–1.2 g of Mg^2+^) in the first 24 h is recommended, followed by 4–6 g/day for 3 or 4 days [92]. For the non-acute setting, oral Mg^2+^ supplementation of 3 × 120 mg/day is more convenient while being sufficiently effective [93]. Parenteral and oral Mg^2+^ supplementation is therefore recommended in many of the hypomagnesemias, including Gitelman syndrome, Bartter syndrome, EAST syndrome and those caused by *TRPM6* mutations [14, 31, 41]. The resulting rise in serum Mg^2+^ concentration often alleviates symptoms, such as seizures and secondary hypocalcemia, even though normal Mg^2+^ values are rarely reached [14, 75]. Further correction of the hypomagnesemia is generally impeded by the gastro-intestinal side effects frequently associated with oral Mg^2+^ supplementation. Paradoxically, higher doses of oral Mg^2+^ might even be detrimental because of the resulting diarrhea [31]. Also, attention should be given to the type of oral Mg^2+^ supplementation given since some preparations have a better bioavailability than others. For this reason, we recommend magnesium chloride or magnesium glycerophospate rather than magnesium oxide or magnesium sulfate for oral Mg^2+^ supplementation [31].

Since hypokalemia is commonly associated with hypomagnesemia, especially in the Gitelman-like hypomagnesemias, one might consider treatment with the epithelial Na^+^ channel ENaC or aldosterone blockers. However, care should be taken, especially in the young child, since this treatment will interfere with the distal compensatory salt reabsorption of the kidney [27]. Lastly, seizures are a known result of several genetic hypomagnesemias, as a primary effect of the genetic defect or secondary to the Mg^2+^ deficiency. In both cases, treatment with anticonvulsants such as valproate or phenobarbital might be beneficial [41].

## Summary and future perspectives

**Fig. 2:**
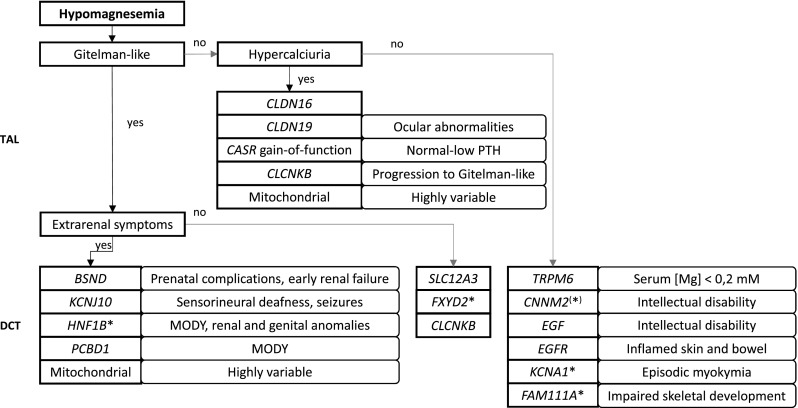
Diagnostic flowchart for a suspected genetic cause of hypomagnesemia. This diagnostic flowchart is primarily provided to give an impression of the clinical characteristics of all known genetic causes of hypomagnesemia. Genetic testing can confirm or reject a diagnosis. *Asterisks* indicate dominantly inherited disorders

In summary, the identification of the different genetic hypomagnesemias has aided our understanding of the important role of the kidney—the TAL and DCT in particular—in maintaining Mg^2+^ homeostasis. Based on the combination of increased fractional renal Mg^2+^ excretion with familial occurrence and a variety of additional symptoms, it is possible to recognize cases with a genetic cause and differentiate between them to a certain extent (see Fig. 2). Subsequent characterization of the affected genes and proteins has improved our understanding of which molecular pathways are involved in Mg^2+^ homeostasis. Hypercalciuric hypomagnesemia is thereby often the presentation of a defect in the TAL, while Gitelman-like and “other” hypomagnesemias are generally localized to the DCT. Treatment still depends on Mg^2+^ supplementation, but an increased understanding of the pathophysiological mechanisms might make discovery of new treatment opportunities possible in the future.

Additional fundamental research might further elucidate the mechanisms of transport and regulation exploited by the DCT and TAL to maintain Mg^2+^ homeostasis. Not only does the basolateral Mg^2+^ transporter need to be identified, but the precise pathophysiological mechanisms by which known mutations cause hypomagnesemia also need further clarification. This even holds true for several well-known and thoroughly described hypomagnesemias, such as Gitelman syndrome. After all, some observations cannot be explained by current understanding. The proposed atrophy of the DCT, for example, has been observed in mice with Gitelman syndrome [29], but it is not present in mice on chronic thiazide treatment-induced hypomagnesemia [30]. The proposed decisive role for the membrane potential of the apical membrane is also not completely satisfying, especially since its role is proven for only some of the genetic hypomagnesemias [78].

On the other hand, some pathways linked to hypomagnesemia might be underestimated. The EGF/EGFR pathway and the insulin and estrogen pathways seem to be of significant importance in terms of increasing Mg^2+^ transport but there is currently little evidence linking them to hypomagnesemic pathology (reviewed in [1]). Additionally, the role of the Na^+^-K^+^-ATPase might be more important than currently appreciated. The Na^+^-K^+^-ATPase is known to occupy a central position in many of the Gitelman-like hypomagnesemia, while it has the potential of being involved in EGFR activation by means of the Na^+^-K^+^-ATPase-Src-kinase complex [94]. The pathophysiology underlying mitochondria-associated diseases might also be of great interest since at least one of the other hypomagnesemias has been reported to be characterized by a vastly diminished size and number of mitochondria in the DCT cells [95]. However, it is not yet clear whether this is a cause, epiphenomenon or result of the hypomagnesemia. Lastly, the recent breakthroughs in identifying new pathways involved in Na^+^ regulation by the DCT [96] could also shed new light on the transport of other ions in this segment. Identification of these other pathways will hopefully provide decisive evidence on the mechanisms of Mg^2+^ reabsorption in the kidney and might open new roads to causal treatment of hypomagnesemia.
